# Supplementary material for: Identification of photoperiod-induced specific miRNAs in the adrenal glands of Sunite sheep (Ovis aries)
Source: Front Vet Sci. 2022 Jul 22;9:888207. doi: 10.3389/fvets.2022.888207 (PMC9354845; doi:10.3389/fvets.2022.888207)
Supplement: Supplementary file 1 [file Data_Sheet_1.ZIP › Supplementary file/miRDeep2 file.zip!/miRDeep2 file/Know_Structure/SPLP42A1/oar-mir-539.pdf]

miRBase precursor : oar-mir-539
Total read count : 1536
oar-miR-539-5p read count : 16
oar-miR-539-3p read count : 1519
remaining reads : 1

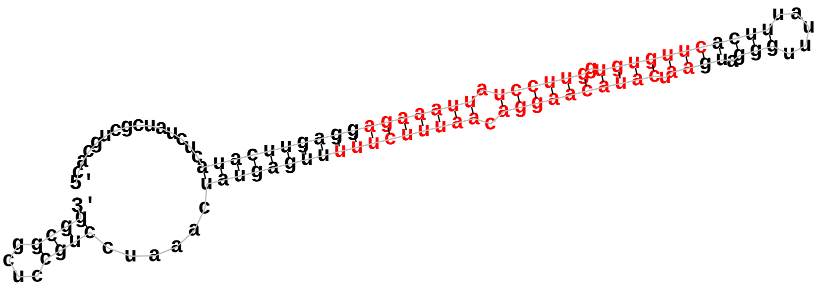

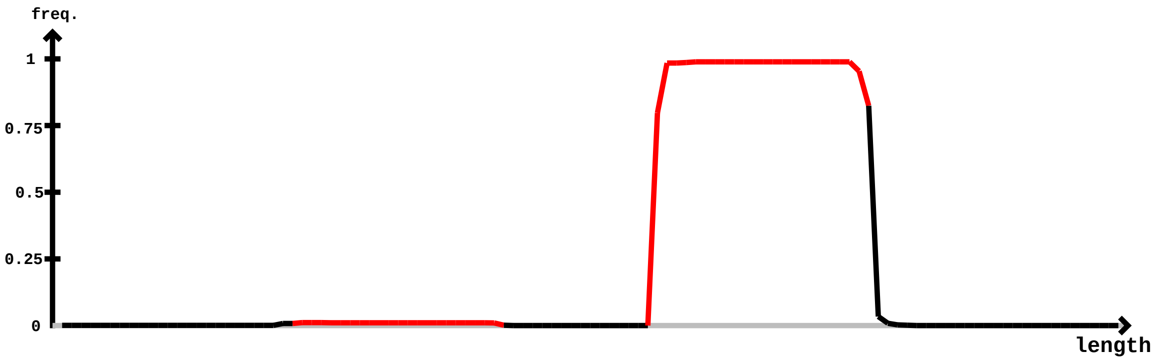


```
                                                                              oar-miR-539-3p
                                oar-miR-539-5p
5'- cacgucgcuaucucauacuugaggagaaauuauccuugguguguucacuuuauuugggaugaaucauacaaggacaauuucuuuuugaguaucaaauccugccucggcgg   -3'    exp
    ..............(((((((((((((((((.((((((.(((((((((((.....))).)))).)))))))))).))))))))))))))))).......((((....))))     reads     mm      sample
    cacgucgcuaucucauacuugaggaga...................................................................................     1         0       seq
    ......................ggagaaauuauccuuggugugu..................................................................     1         0       seq
    ......................ggagaaauuauccuugguguguu.................................................................     10        0       seq
    ........................agaaauuauccuugguguguu.................................................................     3         0       seq
    ........................agaaauuauccuugguguguuc................................................................     2         0       seq
    .............................................................aaucauacaaggacaauuucu............................     53        0       seq
    .............................................................aaucauacaaggacaauuucuu...........................     187       0       seq
    .............................................................aaucauacaaggacaauuucuuu..........................     968       0       seq
    .............................................................aaucauacaaggacaauuucuuuu.........................     16        0       seq
    .............................................................aaucauacaaggacaauuucuuuuu........................     1         0       seq
    ..............................................................aucauacaaggacaauuucu............................     1         0       seq
    ..............................................................aucauacaaggacaauuucuu...........................     12        0       seq
    ..............................................................aucauacaaggacaauuucuuu..........................     246       0       seq
    ..............................................................aucauacaaggacaauuucuuuu.........................     22        0       seq
    ..............................................................aucauacaaggacaauuucuuuuu........................     6         0       seq
    ................................................................cauacaaggacaauuucuuuu.........................     1         0       seq
    ................................................................cauacaaggacaauuucuuuuu........................     2         0       seq
    .................................................................auacaaggacaauuucuuuuug.......................     2         0       seq
    .................................................................auacaaggacaauuucuuuuuga......................     2         0       seq
```
